# Reduction in BMI z-score and improvement in cardiometabolic risk factors in obese children and adolescents. The Oslo Adiposity Intervention Study - a hospital/public health nurse combined treatment 

**DOI:** 10.1186/1471-2431-11-47

**Published:** 2011-05-27

**Authors:** Magnhild L Pollestad Kolsgaard, Geir Joner, Cathrine Brunborg, Sigmund A Anderssen, Serena Tonstad, Lene Frost Andersen

**Affiliations:** 1 Department of Pediatrics, Oslo University Hospital, Ullevål, PB 4956 Nydalen, 0424 Oslo, Norway; 2 Department of Health Management and Health Economics, Institute of Health and Society, University of Oslo, Oslo, Norway; 3 Centre for Clinical Research, Unit of Epidemiology and Biostatistics, Oslo University Hospital, Oslo, Norway; 4 Department of Sports Medicine, The Norwegian School of Sport Sciences, Oslo, Norway; 5 Department of Preventive Cardiology, Oslo University Hospital, Oslo, Norway; 6 Department of Nutrition, Institute of Basic Medical Sciences, University of Oslo, Oslo, Norway

## Abstract

**Background:**

Weight loss and increased physical fitness are established approaches to reduce cardiovascular risk factors. We studied the reduction in BMI z-score associated with improvement in cardiometabolic risk factors in overweight and obese children and adolescents treated with a combined hospital/public health nurse model. We also examined how aerobic fitness influenced the results.

**Methods:**

From 2004-2007, 307 overweight and obese children and adolescents aged 7-17 years were referred to an outpatient hospital pediatrics clinic and evaluated by a multidisciplinary team. Together with family members, they were counseled regarding diet and physical activity at biannual clinic visits. Visits with the public health nurse at local schools or at maternal and child health centres were scheduled between the hospital consultations. Fasting blood samples were taken at baseline and after one year, and aerobic fitness (VO_2peak_) was measured. In the analyses, 230 subjects completing one year of follow-up by December 2008 were divided into four groups according to changes in BMI z-score: Group 1: decrease in BMI z-score≥0.23, Group 2: decrease in BMI z-score≥0.1-< 0.23, Group 3: decrease in/stable BMI z-score≥0.0-< 0.1, Group 4: increase in BMI z-score (>0.00-0.55).

**Results:**

230 participants were included in the analyses (75%). Mean (SD) BMI z-score was reduced from 2.18 (0.30) to 2.05 (0.39) (p < 0.001) in the group as a whole. After adjustment for BMI z-score, waist circumference and gender, the three groups with reduced BMI z-score had a significantly greater reduction in HOMA-IR, insulin, total cholesterol, LDL cholesterol and total/HDL cholesterol ratio than the group with increased BMI z-score. Adding change in aerobic fitness to the model had little influence on the results. Even a very small reduction in BMI z-score (group 3) was associated with significantly lower insulin, total cholesterol, LDL and total/HDL cholesterol ratio. The group with the largest reduction in BMI z-score had improvements in HOMA-IR and aerobic fitness as well. An increase in BMI z-score was associated with worsening of C-peptide and total/HDL cholesterol ratio.

**Conclusions:**

Even a modest reduction in BMI z-score after one year of combined hospital/and public health nurse intervention was associated with improvement in several cardiovascular risk factors.

## Background

The prevalence of overweight and obesity among children is a major problem in public health [1]. It is estimated that approximately 15-20% of children and adolescents in Norway are overweight or obese [2-4]. Childhood obesity is associated with a clustering of cardiovascular risk factors as hypertension, dyslipidemia and impaired glucose tolerance [5,6], and increases the risk for adult cardiovascular disease [7]. In children aged ≥3 years obesity is an increasing important predictor of adult obesity [8].

Treatment for children who are overweight or obese has focused on a variety, as well as a combination, of interventions that include dietary modifications, increased physical activity, and restructuring of eating behaviors [9]. Treatment of children seems to be more effective than treatment of adults [10], but it is not clear which treatment program is the most effective [9,11,12]. Reduction of overweight will most likely not be achieved in children receiving no treatment [13].

Several studies show that reduction in body weight in childhood due to lifestyle intervention improves cardiovascular risk factors in obese children [14-23]. In adults it is well established that a 5-10% weight reduction is enough to improve several cardiovascular risk factors [24]. However, only a few studies have investigated the reduction in BMI z-score necessary to improve cardiovascular risk factors in obese children and adolescents, and this question deserves further attention [25-28].

Both fatness and fitness are shown to have associations with cardiovascular risk factors in children, and aerobic fitness might reduce the negative effect of obesity on these risk factors in children [29-31]. It might therefore be important to consider both fatness and aerobic fitness when studying cardiovascular risk factors in children.

The purpose of the present study was to identify the magnitude of reduction in BMI z-score associated with improvement in cardiometabolic risk factors in overweight and obese children and adolescents. We also examined how aerobic fitness influenced the relation between change in BMI z-score and change in risk factors.

## Methods

### Intervention

In 2004 the "Oslo Adiposity Intervention Study", a long term intervention program for weight loss, was initiated by Department of Pediatrics, Oslo University Hospital. Children or adolescents aged 3-17 years, residing in the city of Oslo, referred by a pediatrician, general practitioner or public health nurse and with a body weight above the 97.5 percentile for height according to Norwegian percentiles [32] were eligible. The study was approved by the Norwegian Data Inspectorate and evaluated by the Regional Committee for Medical and Health Research Ethics. Written informed consent was obtained from at least one of the parents, and all children above 12 years of age.

The program was designed to encourage sustained lifestyle changes in the participants and their families. At the start of the program (baseline) the participants and one or both parents met a pediatrician and a clinical nutritionist separately, while at the follow-up visits they met both members of the treatment team at the same time. The follow-up visits were scheduled after approximately 6 months and one year. Each of these visits lasted about one hour. For immigrant participants and families interpreters were used when needed. During the visit, a medical history was recorded and the participants underwent a physical examination, and together with family members were counseled regarding diet and physical activity. The clinical nutritionist focused on meal planning, portion sizes, increasing fruit and vegetables intake, using more whole grain food, choosing low fat dairy products, avoiding sugar containing drinks, and limiting sweets to once a week. Participants were encouraged to engage in at least 60 minutes of physical activity (walking, swimming, organized sports etc) during the day in accordance with official health recommendations [33]. The participants were also encouraged to limit TV watching and PC activities. We also discussed with the parents how they could facilitate the desirable lifestyle behaviors. Voluntary physical activity groups (swimming, active games) were directed by experienced instructors who emphasized the enjoyable rather than competitive aspects of the activity. In addition, 29 subjects took part in a project involving 60 minutes of biweekly play for 5 months, which is described in details elsewhere [34].

After every visit at the hospital clinic a copy of the child's clinical notes was sent to the public health nurse informing about the child's condition, progress and recommendations. Nurses were located at local schools or maternal and child health centres. The nurse was in an accompanying letter asked to have contact with the participants and families in between the hospital consultations. Approximately 80% of the children/families stated that they had at least one contact with the public health nurse during the one year follow-up. The role of the public health nurses was to motivate the participants between the hospital consultations. On occasion, they also measured the child's weight and height. Twice a year the public health nurses were invited to meet the hospital staff to discuss common problems and receive guidance. In December 2006 60 public health nurses who had treated in total 136 participants in the period 2004-2006 completed an evaluation form. On average they reported meeting each participant once a month (ranging from two times a year to three to four times a month) and most of them (> 80%) also reported counseling one or both parents, either by personal meeting or by telephone.

### Subjects

From February 2004 until December 2007, 307 children and adolescents aged 7-17 years were included in the study, and of these 246 subjects completed the one year follow-up by 31.12.08. There were 52 subjects (16.9%) who dropped out before the one year follow-up, while nine subjects did not complete the one year follow-up before 31.12.08.

Of the 246 subjects who completed the one year follow-up twelve subjects were excluded, so of the total sample 230 (75%) were included in the data analyses. The twelve subjects were excluded for the following reasons: two subjects joined another organized obesity treatment program, one subject was diagnosed with Cushing's syndrome, one subject was diagnosed with Crohn's disease and eight subjects started medication that could influence their body weight and/or glucose metabolism (metformin, sibutramine, orlistat or methylphenidate). Most of the participants (91.5%, n = 214) completed the one-year follow-up 10-15 months after baseline. In the case of four children, 19-26 months passed between baseline and the one-year follow-up. These four subjects were excluded from the analyses. Of the 230 subjects included in the present analyses, approximately 44% were of European ethnicity (39% of Norwegian ethnicity), and 46% were first or second-generation immigrants, from Asia (35%) (included Turkey), Africa (8%) and South America 3%. The remaining 10% were mixed (mostly Asia/Europe).

### Anthropometry

Height was measured to the nearest 1 mm with a stadiometer (Seca 222, Germany). Body weight was measured with the subject dressed in light clothing to the nearest 0.1 kg using a mobile digital scale (Seca 720). BMI was calculated and each BMI value was standardized by conversion to a z-score (BMI z-score) in groups defined by age and gender, using the Centers for Disease Control and Prevention (CDC) growth charts 2000 [35]. This calculation was done using EpiInfo 2005 version 3.3.2. The children and adolescents were categorized as overweight or obese according to the age- and gender-specific definition of the International Obesity Task Force (IOTF) [36]. The term iso-BMI30 is used to refer to the IOTF BMI value corresponding to an adult BMI of 30 kg/m^2^. This term was introduced by Prof Claude Marcus, Karolinska University Hospital, Sweden. We also used the crude BMI value of the participants and subtracted the iso-BMI30 value, designated as ΔIsoBMI30 (Δiso-BMI30 = actual BMI - iso-BMI30).

We defined successful treatment as no increase in BMI z-score in accordance with Nowicka et al [37]. The 230 children included in the analyses were divided into groups according to changes in BMI z-score. The subjects with a decrease in BMI z-score (including seven subjects that had an unchanged BMI z-score) were grouped separately from participants with an increase in BMI z-score. Thus, we formed four groups: group 1 with a decrease in BMI z-score ≥ 0.23 (n = 59); group 2 with a decrease in BMI z-score ≥0.10-< 0.23 (n = 63); group 3 with a decrease in/stable BMI z-score ≥ 0.00-< 0.10 (n = 61); and group 4 with an increase in BMI z-score > 0.00-0.55 (n = 47).

### Pubertal stage

A pediatrician assessed pubertal stage according to a two-point scale as 1) pre-pubertal stage (including Tanner stage 1) and 2) ongoing maturation or mature state of development (including Tanner stage 2, 3, 4 and 5). Girls were staged according to breast development and pubic hair growth and boys were staged according to pubic hair growth and male genital stages [38].

### Blood samples

Fasting blood samples were taken in the morning between 8:00 and 11:00 am at baseline and at the one year follow-up. Total cholesterol, high-density lipoprotein (HDL) cholesterol, low-density lipoprotein (LDL) cholesterol, triglycerides and glucose concentrations were analyzed with absorbance photometry (Cobas Integra 800, Roche Diagnostics, Mannheim, Germany). HbA1c was analyzed with high performance liquid chromatography (HPLC) (Tosho GT), and insulin and C-peptide were analyzed by a noncompetitive immunofluorometric assay (DELFIA kit from Wallac OY, Turku, Finland).

We used the homoeostasis model assessment for insulin resistance (HOMA-IR) to estimate insulin resistance [39]. HOMA-IR was calculated from the following formulas [39]; HOMA-IR = (insulin in mU/L (mU/L = pmol/L/6) × glucose in mmol/L)/22.5, thus, higher values of HOMA-IR representing greater degrees of insulin resistance.

### Aerobic fitness

At baseline participants were invited to undergo an aerobic fitness test on a treadmill. The aerobic fitness test was offered again every 6 to 12 months. Aerobic fitness defined as (VO_2peak_) ml·kg^-1^·min^-1 ^was performed on a Woodway treadmill (USA), using the Oslo protocol [40]. The work load (speed and inclination) was increased until exhaustion. Gas exchange and ventilatory variables were measured during running using the Sensor Medics, Vmax Spektra (Yorba Linda, CA, USA). Achievement of VO_2peak _was based on a subjective assessment that the participant had reached his or her maximal effort and was unwilling to continue despite extensive encouragement from the test leaders. A total of 112 participants completed the fitness test both at baseline and after one year; however, ten test results were not satisfactory leaving 102 subjects included in the aerobic fitness analyses.

### Statistical analyses

Baseline demographical, clinical and metabolic data separated according to reduction in BMI z-score are presented as means with SD, medians with 25^th ^percentiles and 75^th ^percentiles or percentages, see Additional files 1 and 2. The Chi-square test was used for categorical variables, One-Way ANOVA was used for normally distributed variables, and Kruskal-Wallis for non-normally distributed variables. When comparing the changes in metabolic parameters between the 4 groups One-Way ANOVA was used and linear regression was used to adjust for baseline BMI z-score, waist circumference, gender and aerobic fitness. An investigation of the correlation between the potential confounders was performed before multivariate analysis. The highest correlation was between BMI z-score and waist circumference (0.55), but since this is below 0.8 level there should not be a risk of collinearity [41]. Paired sample t-tests were used to assess one-year changes within each of the four groups, see Additional file 3.

All p-values are two-sided and a 5% level of significance was used [42]. The statistical analyses were performed with SPSS 15.0 (SPSS Inc., Chicago, IL).

## Results

### Baseline characteristics

Baseline demographical, clinical and metabolic data separated according change in BMI z-score are shown in Additional files 1 and 2. The four groups differed according to gender, weight, waist circumference, BMI, ΔIsoBMI30 and BMI z-score, see Additional file 1. At baseline the group with the largest reduction in BMI z-score (Group 1) had significantly lower weight, waist circumference, BMI, ΔIsoBMI30 and BMI z-score than the three other groups (data not shown). C-peptide, triglycerides and aerobic fitness at baseline also differed between the groups, but the other eight metabolic parameters did not differ between the groups, see Additional file 2. The group with the largest reduction in BMI z-score (Group 1) had significantly higher aerobic fitness and significantly lower C-peptide concentration than the three other groups (data not shown).

At baseline there were no differences between the completers (n = 230) and dropouts (n = 52) with respect to ethnicity (p = 0.13), BMI z-score (p = 0.08), ΔIsoBMI30 (p = 0.89), BMI (p = 0.18) or the metabolic parameters studied (all p-values < 0.05). The mean BMI z-score among the dropouts was 2.10 (range 1.29-2.76) versus 2.18 (range 1.30-3.17) for completers. The dropouts were significantly older (12.8 years, range 7.0-17.2) than the completers (11.4, range 7.0-17.6) (p < 0.001).

### Changes in metabolic parameters and aerobic activity according to changes in BMI z-score after one year intervention

After one year there were statistically significant reductions in BMI z-score (mean reduction -0.13, p < 0.001) and ΔIsoBMI30 (mean reduction -0.9, p < 0.001) in the total group. We also found significant improvements in HOMA-IR, insulin, total cholesterol, LDL cholesterol and total cholesterol/HDL cholesterol ratio in the total group (data not shown). Of the total group, 176 (76.5%) decreased their BMI z-score, seven (3%) had a stable BMI z-score and 47 (20.5%) increased their BMI z-score during the intervention period.

The three groups with reduced BMI z-score had a significantly larger reduction in HOMA-IR, insulin, LDL cholesterol and total cholesterol/HDL cholesterol ratio than the group with increased BMI z-score, see Additional file 3. When adjusting for gender, baseline BMI z-score and waist circumference the three groups with reduced BMI z-score also had a significantly larger reduction in total cholesterol than the group with increased BMI z-score. In the 102 subjects with aerobic fitness measurement we also adjusted for improvement in aerobic fitness in addition to gender, baseline BMI z-score and waist circumference. We found that the three groups with reduced BMI z-score still had a significantly larger reduction in HOMA-IR, insulin and total cholesterol than the group with increased BMI z-score (data not shown). According to total cholesterol/HDL cholesterol the significant difference between the group with increase in BMI z-score and group 2 (decrease in BMI z-score ≥0.1-< 0.23) disappeared. The difference in plasma LDL between groups became non significant (p = 0.06) after adjusting for fitness.

A very small reduction in BMI z-score after one year follow-up (group 3) was associated with significantly lower insulin, total cholesterol, LDL and total cholesterol/HDL cholesterol ratio, see Additional file 3. The improvement in HOMA-IR was of borderline significance in this group. Only the group with the largest reduction in BMI z-score (group 1) had significant improvement in aerobic fitness, see Additional file 3. The group with an increase in BMI z-score (group 4) had a significant increase in C-peptide and total cholesterol/HDL cholesterol ratio after the intervention.

## Discussion

The overall study results showed that after one year follow-up in the "Oslo Adiposity Intervention Study" there were a statistically significant reduction in BMI z-score and Δiso-BMI30 in the total group. We found that even a stable/modest reduction in BMI z-score (≥ 0.00-< 0.10) was associated with improvement in several cardiovascular risk factors. An increase in BMI z-score was associated with worsening of some of the risk factors studied.

The participants in our study had a mean reduction in BMI z-score of 0.13. In comparison Oude et al reported reductions in BMI z-score after one year of lifestyle interventions between 0.17 to 0.24 in children younger than 12 years and from 0.08 to 0.21 in children older than 12 years [12]. The group with the greatest reduction in BMI z-score in our study tended to be the youngest, but there were no significant difference between the four groups. Other studies have shown that age might be an important factor for success, with younger children achieving larger reduction in BMI z-score than older ones [43-45]. The reduction in our study is of the same magnitude as two Swedish studies [37,46]. Reinehr et al found a mean reduction in BMI-SDS of 0.36 after one year among children and adolescents attending their obesity intervention (Obeldick) [43]. This reduction is larger than in our study, but only children and adolescents motivated for lifestyle intervention were included in their study [43] in contrast to the present study, where motivation was not assessed.

The group in our study with the greatest reduction in BMI z-score was the group with the lowest baseline BMI z-score. This is in agreement with other investigators [28]. The group in our study with the lowest BMI z-score initially also tended to have the lowest HOMA-IR and insulin values at the beginning of the intervention even though the difference was not statistically significant. Sabin et al [45] saw a trend towards greater improvement in BMI SDS over one year in those with initially lower HOMA-IR, although the differences between the groups were not statistically significant.

We found that a very small reduction in BMI z-score (≥ 0.00-< 0.10) improved insulin and insulin resistance. This is an important finding since insulin resistance may underlie future risk of diabetes and cardiovascular disease [47]. In comparison, Reinehr et al found a significant improvement in insulin sensitivity expressed as ISI-HOMA with reduction in SDS-BMI ≥0.5 after one year follow-up [25]. The authors suggested that insulin sensitivity may improve significantly at a lower level of weight reduction in larger cohorts, compared to their small cohort (n = 57) [25]. They found the same results in another study using HOMA as measurement for insulin resistance [26]. Ford et al [28] found an improvement in HOMA-IR with a reduction in BMI z-score ≥0.25 among 88 obese adolescents, but greater benefits occurred with a reduction ≥0.5. Unlike Reinehr et al we did not find an increase in insulin resistance in the group with an increase in BMI z-score [25,26], though C-peptide concentrations increased, indicating increased insulin production and future risk of diabetes. None of the groups showed significant improvements in glucose concentrations. This is in agreement with other studies [14,25]. Reinehr et al found that glucose concentration did not improve in the group with a BMI z-score reduction as large as ≥0.5 [25]. No change in glucose and a simultaneous lowering of insulin indicates that the insulin resistance is improved, and less insulin is needed to maintain the same glucose concentration.

We found a small but significant improvement in total cholesterol, total cholesterol/HDL cholesterol ratio and LDL cholesterol concentrations in the total intervention group. Also other studies evaluating intervention programs for childhood obesity have found improvements in lipid profile [14,22,26,27]. In a study investigating changes in the atherogenic risk factor profile according to degree of weight loss, Reinehr et al found that reduction in SDS-BMI between 0.25 and 0.5 was associated with a significant improvement in LDL cholesterol, but not in triglycerides and HDL cholesterol [26]. When the reduction in SDS-BMI was ≥0.5 they found significant improvements in all lipid fractions studied [26]. Another study found improvements in LDL cholesterol, total cholesterol/HDL cholesterol and triglycerides with reduction in SDS-BMI ≥0.25 [28]. None of the groups in our study showed significant improvements in HDL cholesterol and triglycerides, but total cholesterol, LDL cholesterol and total cholesterol/HDL cholesterol improved even in the group with modestly improved or stable BMI z-score (≥ 0.00-< 0.10). If maintained, these improvements may impact the atherosclerotic process which begins in childhood and is causally linked to blood cholesterol levels [48]. The group with increase in BMI z-score also experienced an increase in total cholesterol/HDL cholesterol ratio.

When comparing changes in metabolic parameters between the three groups with stable or reduced BMI z-score and the group with increased BMI z-score adjustment was made for improvement in aerobic fitness where the data was available, since cardiovascular fitness and CVD risk factors might be independently related in obese children [49]. A previously published study found that low cardiovascular fitness was strongly associated with the clustering of CVD risk factors in children independent of country, age and sex [50]. Adjusting for fitness in our study population had little influence on changes in HOMA-IR, insulin and total cholesterol. Though total/HDL cholesterol and LDL cholesterol effects were somewhat explained by changes in fitness this may be due to the smaller sample size. Only 102 subjects (44%) had data on aerobic fitness, and when repeating the analyzes in the 102 subject only adjusting for gender, baseline BMI z-score and waist circumference we got the same results as when aerobic fitness were included in the model. Former research has demonstrated divergent results regarding the relationship between insulin resistance and physical fitness in obese children and adolescents, and the effect of fitness on insulin may be mediated through a direct pathway and indirectly through changes in body composition [49,51]. A newly published study among 6^th ^grade youths concluded that both fatness and fitness are associated with cardiometabolic risk factors, but that fatness has a stronger association than fitness [52]. This was also the conclusion in a review from Eisenmann where he states that the association between fatness and metabolic syndrome is stronger than for fitness in children and adolescents [53].

Earlier published studies evaluating different treatment approaches for childhood obesity have focused on either hospital based or primary care based treatment. To our best knowledge this is the first study combining hospital and public health nurse approaches. Advantages with involving the public health nurse in the treatment are the opportunity for families to get follow-up in their local environment, and that the public health nurses have good knowledge about preventive work among children and adolescents. A limitation of our study is that we do not have data regarding the frequency of contact between the public health nurse and each subject and their families. One of the strengths of our study is the lack of strict selection criteria. All children and adolescents who were referred and met our inclusion criteria were included in the project regardless of motivational level. Also, we had a good completion rate as > 80% of participants completed the one year follow-up. The participants who did not complete the one year follow-up were older than the completers, but did not differ from completers in regard to BMI z-score or ethnicity. Summerbell et al reported that drop out rates vary from 7-43% in studies of 12 months duration [9]. A limitation of the study is that we do not have a control group of overweight and obese children receiving no treatment. Other studies have, however, shown that overweight and obese children receiving no lifestyle intervention are more likely to increase their overweight [13,27]. Another limitation is that the follow-up in our study was relatively short and maintenance of the treatment effect was not studied beyond one year. Furthermore, we do not have data of good quality regarding dietary habits and physical activity. We measured change in aerobic fitness as ml·kg^-1^·min^-1^. One may argue that the improvements could be due to change in mass (kg) and not "real" aerobic fitness. To avoid this it would have been useful to express aerobic fitness also as mean change in treadmill time to exhaustion or expressed as ml pr unit fat free mass. Unfortunately we did not collect data on time to exhaustion or fat free mass.

## Conclusions

In conclusion even a modest reduction in BMI z-score after one year intervention was associated with improvement in insulin, total-, LDL, and total/HDL cholesterol. An increase in BMI z-score during the one year period was associated with worsening of C-peptide and total/HDL cholesterol. Studies of longer duration are needed to see if the effect of treatment is maintained beyond one year, and whether the improvement in cardiovascular risk factors impacts risk of later cardiovascular morbidity and mortality.

## Abbreviations

BMI: body mass index; HOMA-IR: homoeostasis model assessment for insulin resistance; HDL: high-density lipoprotein; LDL: low-density lipoprotein; IOTF: International Obesity Task Force

## Competing interests

The authors declare that they have no competing interests.

## Authors' contributions

MLPK was responsible for patient enrollment and participated in the design of the study. She performed the statistical analysis, interpreted the data and wrote the manuscript. LFA, GJ and ST participated in the design of the study, contributed interpreting the data and revising successive drafts of the manuscript. CB helped with the statistical analysis and revising the manuscript. SAA contributed in deciding method of testing aerobic fitness and revising the manuscript. All authors read and approved the final manuscript.

## Pre-publication history

The pre-publication history for this paper can be accessed here:

http://www.biomedcentral.com/1471-2431/11/47/prepub

## Supplementary Material

Additional file 1 **Table 1. Baseline characteristics of the subjects (n = 230) separated according to change in BMI z-score**. Table showing means with standard deviations (SD), median (25th,75th percentiles) or percentages Click here for file

Additional file 2 **Table 2. Baseline metabolic characteristics of the subjects separated according to change in BMI z-score**. Table showing means with standard deviations (SD) or median (25th,75th percentiles) Click here for file

Additional file 3 **Table 3. Changes in cardiovascular risk factors after one year follow- up according to changes in BMI z-score**. Table showing means with standard deviations (SD) Click here for file
