# Correction: “Reduction in BMI z-score and improvement in cardiometabolic risk factors in obese children and adolescents. The Oslo adiposity intervention study - a hospital/public health nurse combined treatment.”

**DOI:** 10.1186/1471-2431-12-77

**Published:** 2012-06-18

**Authors:** Magnhild L Pollestad Kolsgaard, Geir Joner, Cathrine Brunborg, Sigmund A Anderssen, Serena Tonstad, Lene Frost Andersen

**Affiliations:** 1 Department of Pediatrics, Oslo University Hospital, Ullevål, PB 4956, Nydalen 0424, Oslo, Norway; 2 Department of Health Management and Health Economics, Institute of Health and Society, University of Oslo, Oslo, Norway; 3 Centre for Clinical Research, Unit of Epidemiology and Biostatistics, Oslo University Hospital, Oslo, Norway; 4 Department of Sports Medicine, The Norwegian School of Sport Sciences, Oslo, Norway; 5 Department of Preventive Cardiology, Oslo University Hospital, Oslo, Norway; 6 Department of Nutrition, Institute of Basic Medical Sciences, University of Oslo, Oslo, Norway

## **Correction**

After publication of this work [1], it came to our attention that the laboratory we use changed the method of analysis for insulin and C-peptide during our data collection. Before March 2007 the method used for insulin was competitive radioimmunoassay (Linco Research Inc, St Charles, MO, USA) and for C-peptide competitive luminoimmunoassay (Immulite 2000, Diagnostic Products Corporation, CA, USA). After March 2007 noncompetitive immunofluorometric assays (DELFIA kit form Wallac OY, Turku, Finland) were used.

We have therefore recalculated the insulin and C-peptide values analyzed after March 2007, and repeated the statistical analyses involving insulin, C-peptide and HOMA-IR.

Corrections in the Abstract

In **Results** the fifth sentence should have read:

“Even a very small reduction in BMI z-score (group 3) was associated with significantly lower total cholesterol, LDL cholesterol and total/HDL cholesterol ratio.”

Explanation:

After recalculation the significant reduction in insulin in the group with a very small reduction in BMI z-score disappeared. None of the groups had significant improvements in insulin concentration (the improvements in group 1 and 2 were of borderline significance).

The seventh sentence should have read:

“An increase in BMI z-score was associated with a worsening of HOMA-IR, insulin, C-peptide and total/HDL cholesterol ratio.”

Explanation:

The group with an increase in BMI z-score had a worsening in HOMA-IR and insulin in addition to C-peptide and total/HDL cholesterol ratio as previously reported.

Corrections in the Main manuscript

## **Results**

In the section **“Baseline characteristics”** on page 4

The fourth-fifth sentence should have read:

“Insulin, C-peptide, triglycerides and aerobic fitness at baseline also differed between the groups, but the other seven parameters did not differ between the groups, see corrected version of Additional file 1. The group with the largest reduction in BMI z-score (Group 1) had significantly higher aerobic fitness and significantly lower insulin and C-peptide concentration than the three other groups (data not shown).”

Explanation:

Insulin should be added to the list with metabolic parameters that differed between the groups at baseline. Moreover the group with the largest reduction in BMI z-score (Group 1) had significant lower insulin in addition to lower C-peptide and higher aerobic fitness as previously reported.

In the section **“Changes in metabolic parameters and aerobic activity according to changes in BMI z-score after one year intervention”** on page 4

The second sentence should have read:

“We also found significant improvements in total cholesterol, LDL cholesterol and total cholesterol/HDL cholesterol ratio in the total group (data not shown).”

Explanation:

The significant reduction in HOMA-IR and insulin after one year intervention in the total group disappear.

The tenth sentence (start of paragraph 3) in the same section should have read:

“A very small reduction in BMI z-score after one year follow-up (group 3) was associated with significantly lower total cholesterol, LDL and total cholesterol/HDL cholesterol ratio, see corrected version of Additional file 2.

Explanation:

The significant reduction in insulin in the group with a very small reduction in BMI z-score disappeared. None of the groups had significant improvements in insulin concentration (the improvements in group 1 and 2 were of borderline significance).

The eleventh sentence (paragraph 3) in the same section

The phrase “The improvement in HOMA-IR was of borderline significance in this group” should be deleted.

Explanation:

The improvement in HOMA-IR was no longer borderline significant in the group with the smallest reduction in BMI z-score (group 3).

The thirteenth sentence (paragraph 3) in the same section should have read:

“The group with an increase in BMI z-score (group 4) had a significant increase in HOMA-IR, insulin, C-peptide and total cholesterol/HDL cholesterol ratio after the intervention.”

Explanation:

The group with an increase in BMI z-score (group 4) got a worsening in HOMA-IR and insulin in addition to C-peptide and total/HDL cholesterol ratio as we previously reported.

## **Discussion**

The thirteenth sentence (paragraph three) on page 5 should have read:

“The group in our study with the lowest BMI z-score initially also had the lowest insulin value at the beginning of the intervention. This group also tended to have the lowest HOMA-IR value at baseline even though the difference was not statistically significant.”

Explanation:

We have written that the group in our study with the lowest BMI z-score initially (that is the same group that reduced their BMI z-score the most, group 1) also tended to have the lowest HOMA-IR and insulin values at the beginning of the intervention even though the difference was not statistically significant. The difference between group 1 and the three other groups according to insulin now become statistically significant.

The fifteenth sentence (start of paragraph four) on page 5 should have read:

“We found that a reduction in BMI z-score ≥0.23 (group 4) improved insulin resistance.”

Explanation:

It is no longer correct that the group with a very small reduction in BMI z-score (≥0.00- < 0.10) improved insulin and insulin resistance. Insulin resistance only improved in the group with the largest reduction in BMI z-score, and the improvement in insulin was of borderline significance in this group.

The twenty-first sentence (paragraph four) on page 5 should have read:

“Like Reinehr et al we found an increase in insulin resistance in the group with an increase in BMI z-score [3,4]”.

Explanation:

The group with an increase in BMI z-score now got a worsening in insulin resistance, like Reinehr earlier has reported. The last part of the sentence “though C-peptide concentrations increased, indicating increased insulin production and future risk of diabetes” is no longer relevant for our findings.

The twenty-fifth sentence (end of paragraph four) on page 5 “No change in glucose and a simultaneous lowering of insulin indicates that the insulin resistance is improved, and less insulin is needed to maintain the same glucose concentration” should be deleted.

Explanation:

This is no longer relevant for our findings since insulin did not improve in any of the groups.

## **Conclusions**

The first and second sentences in the conclusion should have read:

“In conclusion even a modest reduction in BMI z-score after one year intervention was associated with improvement in total-, LDL, and total/HDL cholesterol. An increase in BMI z-score during the one year period was associated with worsening of HOMA-IR, insulin, C-peptide and total/HDL cholesterol.”

Explanation:

The improvement in insulin in the group with the small reduction in BMI z-score disappeared (group 3) and the group with increased BMI z-score had an worsening of HOMA-IR and insulin in addition to C-peptide and total/HDL cholesterol

## Pre-publication history

The pre-publication history for this paper can be accessed here:

http://www.biomedcentral.com/1471-2431/12/77/prepub

## Supplementary Material

Additional file 1 **Corrected Table 1.** The values that changed are shown in bold. Click here for file

Additional file 2 **Corrected Table 2.** The values that changed are shown in bold. Click here for file
