# Supplementary material for: Reduction in BMI z-score and improvement in cardiometabolic risk factors in obese children and adolescents. The Oslo Adiposity Intervention Study - a hospital/public health nurse combined treatment
Source: BMC Pediatr. 2011 May 27;11:47. doi: 10.1186/1471-2431-11-47 (PMC3121603; doi:10.1186/1471-2431-11-47)
Supplement: Additional file 1 — Table 1. Baseline characteristics of the subjects (n = 230) separated according to change in BMI z-score. Table showing means with standard deviations (SD), median (25th,75th percentiles) or percentages [file 1471-2431-11-47-S1.PDF]

Table 1: Baseline characteristics of the subjects (n=230) separated according to change in BMI z-score

Table showing means with standard deviations (SD), median (25th,75th percentiles) or percentages

	n	**Group 1** Decrease in BMI z-score ≥0.23	n	**Group 2** Decrease in BMI z-score ≥0.1-<0.23	n	**Group 3** Decrease in/stable BMI z-score ≥0.0-<0.1	n	**Group 4** Increase in BMI z-score >0.00-0.55	*p-value for difference between groups
Age (years)	59	10.9 (2.6)	63	11.3 (2.5)	61	11.4 (2.4)	47	12.1 (2.1)	0.1
Gender									
Boys	30	50.8	20	31.7	32	52.5	27	57.4	0.03
Girls	29	49.2	43	68.3	29	47.5	20	42.6	
Puberty									
Prepubertal	32	55.2	30	47.6	30	50.8	23	52.3	0.87
Pubertal	26	44.8	33	52.4	29	49.2	21	47.7	
Ethnicity									
Ethnic Norwegians	28	47.5	25	39.7	22	36.1	14	29.8	0.3
Other Ethnicities [1]	31	52.5	38	60.3	39	63.9	33	70.2	
Weight (kg)	59	58.0 (46.4 , 74.0)	63	68.5 (55.0 , 81.2)	61	71.2 (56.3 , 89.8)	47	69.9 (59.5 , 86.3)	0.006
Height (cm)	59	150.5 (14.5)	63	151.7 (12.7)	61	154.5 (15.5)	47	156.7 (11.6)	0.1
Waist circumference (cm)	59	79.4 (9.8)	63	85.2 (11.2)	60	88.4 (13.8)	46	87.5 (12.4)	< 0.001
BMI (kg/m2)	59	26.1 (23.4 , 28.7)	63	29.1 (25.8 , 31.7)	61	30.3 (26.9 , 32.6)	47	29.1 (26.6 , 31.5)	< 0.001
Δiso-BMI > 30 [2]	59	1.7 (2.2)	63	4.4 (4.0)	61	5.1 (4.2)	47	3.9 (4.9)	< 0.001
BMI z-score [3]	59	2.0 (0.3)	63	2.2 (0.3)	61	2.3 (0.3)	47	2.2 (0.3)	< 0.001

* Chi-square test for contingency tables, One Way Anova normally distributed variables and Kruskal-Wallis non-normally distributed variables

[1] Mostly from Asia (included Turkey) and Africa

[2] Body mass index units above the BMI corresponding to 30 at age 18 according to the IOTF criteria

[3] Defined by age and gender, using the Centers for Disease Control and Prevention (CDC) growth charts 2000
