# Supplementary material for: Reduction in BMI z-score and improvement in cardiometabolic risk factors in obese children and adolescents. The Oslo Adiposity Intervention Study - a hospital/public health nurse combined treatment
Source: BMC Pediatr. 2011 May 27;11:47. doi: 10.1186/1471-2431-11-47 (PMC3121603; doi:10.1186/1471-2431-11-47)
Supplement: Additional file 2 — Table 2. Baseline metabolic characteristics of the subjects separated according to change in BMI z-score. Table showing means with standard deviations (SD) or median (25th,75th percentiles) [file 1471-2431-11-47-S2.PDF]

Table 2: Baseline metabolic characteristics of the subjects separated according to change in BMI z-score

Table showing means with standard deviations (SD) or median (25th,75th percentiles)

	n	Group 1 Decrease in BMI z-score ≥0.23	n	Group 2 Decrease in BMI z-score ≥0.1-<0.23	n	Group 3 Decrease in/stable BMI z-score ≥0.0-<0.1	n	Group 4 Increase in BMI z-score >0.00-0.55	*p-value for difference between groups
HOMA-IR [1]	49	3.1 (2.0 , 4.1)	54	3.7 (2.0 , 5.7)	48	3.6 (2.5 , 5.9)	41	3.3 (2.4 , 4.4)	0.29
Glucose (mmol/L)	49	4.8 (0.3)	58	4.8 (0.4)	52	4.8 (0.3)	42	4.9 (0.5)	0.75
Insulin (pmol/L)	50	82 (57 , 110)	56	110 (58 , 167)	49	103 (70 , 163)	43	90 (70 , 130)	0.21
HbA1c (%)	49	5.3 (0.3)	60	5.4 (0.3)	51	5.4 (0.3)	44	5.4 (0.3)	0.4
C-peptide	50	666 (310)	56	873 (398)	49	916 (408)	41	865 (349)	0.004
Total cholesterol (mmol/L)	52	4.5 (4.2 , 5.2)	57	4.3 (3.9 , 4.7)	52	4.6 (3.9 , 5.3)	42	4.4 (4.0 , 5.1)	0.11
HDL cholesterol [2] (mmol/L)	52	1.38 (0.29)	57	1.31 (0.32)	52	1.28 (0.29)	42	1.27 (0.23)	0.21
LDL cholesterol [3] (mmol/L)	50	2.91 (0.78)	57	2.61 (0.70)	52	2.82 (0.75)	42	2.65 (0.73)	0.13
Total /HDL cholesterol	52	3.4 (2.9 , 4.1)	57	3.3 (2.8 , 3.9)	52	3.8 (2.9 , 4.5)	42	3.7 (3.1 , 4.3)	0.34
Triglycerides (mmol/L)	52	0.90 (0.67 , 1.34)	57	0.90 (0.61 , 1.23)	52	0.97 (0.76 , 1.51)	42	1.17 (0.81 , 1.65)	0.02
VO_2peak [4] ($ml \cdot kg^{-1} \cdot min^{-1}$)	31	34.7 (4.1)	28	31.5 (3.7)	24	31.2 (4.9)	19	31.8 (5.2)	0.01

*One Way Anova normally distributed variables, Kruskal-Wallis non-normally distributed variables

[1] HOMA-IR: homoeostasis model assessment of insulin resistance, [2] HDL: high density lipoprotein, [3] LDL: low density lipoprotein

[4] VO_2peak: peak oxygen uptake
