# Supplementary material for: Reduction in BMI z-score and improvement in cardiometabolic risk factors in obese children and adolescents. The Oslo Adiposity Intervention Study - a hospital/public health nurse combined treatment
Source: BMC Pediatr. 2011 May 27;11:47. doi: 10.1186/1471-2431-11-47 (PMC3121603; doi:10.1186/1471-2431-11-47)
Supplement: Additional file 3 — Table 3. Changes in cardiovascular risk factors after one year follow- up according to changes in BMI z-score. Table showing means with standard deviations (SD) [file 1471-2431-11-47-S3.PDF]

Table 3: Changes in cardiovascular risk factors after one year follow up according to changes in BMI z-score

Table showing means with standard deviations (SD)

	n	Group 1 Decrease in BMI z-score ≥0.23	n	Group 2 Decrease in BMI z-score ≥0.1-<0.23	n	Group 3 Decrease in/stable BMI z-score ≥0.0-<0.1	n	Group 4 Increase in BMI z-score >0.00-0.55	*p-value for differences between groups	Adjusted** p-value for differences between groups
HOMA-IR	49	-1.1 (2.1) [1, 1a, 2]	54	-1.1 (2.3) [1, 1a, 2]	48	-0.7 (2.6) [1, 1a]	41	0.6 (2.9)	0.003	0.001
Glucose	49	-0.1 (0.3)	58	0 (0.4)	52	0 (0.4)	42	0 (0.4)	0.44	0.48
Insulin	50	-28 (59) [1, 1a, 2]	56	-33 (58) [1, 1a, 2]	49	-19 (66) [1, 1a, 2]	43	13 (78)	0.004	< 0.001
HbA1c	49	0 (0.3)	60	-0.2 (0.3)	51	0 (0.2)	44	0.1 (0.2)	0.15	0.37
C-peptide	50	-47 (236) [1a]	56	-33 (306) [1a]	49	18 (383)	41	110 (235) [3]	0.06	0.02
Total cholesterol	52	-0.4 (0.5) [1, 1a, 2]	57	-0.1 (0.5) [1a]	52	-0.1 (0.5) [1a, 2]	42	0.1 (0.5)	0.002	0.01
HDL cholesterol	52	0.02 (0.23)	57	-0.02 (0.18)	52	0.02 (0.19)	42	-0.04 (0.18)	0.25	0.15
LDL cholesterol	50	-0.35 (0.47) [1, 1a, 2]	57	-0.31 (0.45) [1, 1a, 2]	52	-0.12 (0.42) [1, 1a, 2]	42	0.10 (0.49)	<0.001	< 0.001
Total/HDL cholesterol	52	-0.3 (0.6) [1, 1a, 2]	57	0 (0.6) [1, 1a]	52	-0.2 (0.6) [1, 1a, 2]	42	0.2 (0.5) [3]	<0.001	< 0.001
Triglycerides	52	-0.12 (0.63)	57	0.09 (0.46)	52	-0.09 (0.43)	42	0.03 (0.58)	0.14	0.02
VO_2peak	31	3.0 (5.7) [1, 1a, 2]	28	- 0.1 (4.8)	24	0.7 (5.3)	19	-1.0 (6.2)	0.05	0.02

* One way Anova

** Linear regression. Adjusted for baseline BMI z-score, waist circumference and gender

[1] Means are significant different (p<0.05) from means in group 4. [1a] Means are significant different (p<0.05) from means in group 4 after adjustment.

[2] Significant improvement (p<0.05) after intervention. Paired sample t-test, [3] Significant worsening (p<0.05) after intervention
