# Supplementary material for: Correction: “Reduction in BMI z-score and improvement in cardiometabolic risk factors in obese children and adolescents. The Oslo adiposity intervention study - a hospital/public health nurse combined treatment.”
Source: BMC Pediatr. 2012 Jun 18;12:77. doi: 10.1186/1471-2431-12-77 (PMC3436862; doi:10.1186/1471-2431-12-77)
Supplement: Additional file 2 — Corrected Table 2. The values that changed are shown in bold. [file 1471-2431-12-77-S2.pdf]

**Table 3: Changes in cardiovascular risk factors after one year follow up according to changes in BMI z-score**
Table showing means with standard deviations (SD)

| | n | **Group 1** Decrease in BMI z-score ≥0.23 | n | **Group 2** Decrease in BMI z-score ≥0.1-<0.23 | n | **Group 3** Decrease in/stable BMI z-score ≥0.0-<0.1 | n | **Group 4** Increase in BMI z-score >0.00-0.55 | *p-value for differences between groups | Adjusted** p-value for differences between groups |
|---|---|---|---|---|---|---|---|---|---|---|
| HOMA-IR | 49 | **-0.7** (2.1) [1, 1a, 2] | 54 | **-0.6 (2.6)** [1, 1a] | 48 | **-0.1 (2.7)** [1, 1a] | 41 | **1.4 (2.8)** [3] | **< 0.001** | **< 0.001** |
| Glucose | 49 | -0.1 (0.3) | 58 | 0 (0.4) | 52 | 0 (0.4) | 42 | 0 (0.4) | 0.44 | 0.48 |
| Insulin | 50 | **-16 (57)** [1, 1a] | 56 | **-18 (66)** [1, 1a] | 49 | **-1 (67)** [1, 1a] | 43 | **38 (68)** [3] | **< 0.001** | < 0.001 |
| HbA1c | 49 | 0 (0.3) | 60 | -0.2 (0.3) | 51 | 0 (0.2) | 44 | 0.1 (0.2) | 0.15 | 0.37 |
| C-peptide | 50 | **-46 (233)** [1a] | 56 | **-35 (303)** [1a] | 49 | **16 (380)** | 41 | **104 (232)** [3] | **0.07** | 0.02 |
| Total cholesterol | 52 | -0.4 (0.5) [1, 1a, 2] | 57 | -0.1 (0.5) [1a] | 52 | -0.1 (0.5) [1a, 2] | 42 | 0.1 (0.5) | 0.002 | 0.01 |
| HDL cholesterol | 52 | 0.02 (0.23) | 57 | -0.02 (0.18) | 52 | 0.02 (0.19) | 42 | -0.04 (0.18) | 0.25 | 0.15 |
| LDL cholesterol | 50 | -0.35 (0.47) [1, 1a, 2] | 57 | -0.31 (0.45) [1, 1a, 2] | 52 | -0.12 (0.42) [1, 1a, 2] | 42 | 0.10 (0.49) | < 0.001 | < 0.001 |
| Total/HDL cholesterol | 52 | -0.3 (0.6) [1, 1a, 2] | 57 | 0 (0.6) [1, 1a] | 52 | -0.2 (0.6) [1, 1a, 2] | 42 | 0.2 (0.5) [3] | < 0.001 | < 0.001 |
| Triglycerides | 52 | -0.12 (0.63) | 57 | 0.09 (0.46) | 52 | -0.09 (0.43) | 42 | 0.03 (0.58) | 0.14 | 0.02 |
| $VO_2$peak | 31 | 3.0 (5.7) [1, 1a, 2] | 28 | - 0.1 (4.8) | 24 | 0.7 (5.3) | 19 | -1.0 (6.2) | 0.05 | 0.02 |

* One way Anova
** Linear regression. Adjusted for baseline BMI z-score, waist circumference and gender
[1] Means are significant different (p<0.05) from means in group 4. [1a] Means are significant different (p<0.05) from means in group 4 after adjustment.
[2] Significant improvement (p<0.05) after intervention. Paired sample t-test, [3] Significant worsening (p<0.05) after intervention
